# Are physician-patient communication practices slowly changing in Croatia? – a cross-sectional questionnaire study

**DOI:** 10.3325/cmj.2013.54.185

**Published:** 2013-04

**Authors:** Luka Vučemilo, Marko Ćurković, Milan Milošević, Jadranka Mustajbegović, Ana Borovečki

**Affiliations:** 1 Institute for Ear, Nose and Throat Disease and Head and Neck Surgery, University Hospital Merkur, University of Zagreb School of Medicine, Zagreb, Croatia; 2 University of Zagreb, School of Medicine, Andrija Štampar School of Public Health, Zagreb, Croatia

## Abstract

**Aim:**

To explore physician-patient communication practices during the process of obtaining informed consent in a hospital setting in Croatia.

**Methods:**

Two hundred and fifty patients (response rate 78%) from five tertiary level hospitals in Zagreb, Croatia, anonymously filled in the questionnaire on informed consent and communication practices by Nemcekova et al in the period from April to December 2011.

**Results:**

Eighty five percent of patients received complete, understandable information, presented in a considerate manner. Patients in surgical departments received a higher level of information than those in internal medicine departments. Patients were informed about health risks of the proposed treatments (in 74% of cases) and procedures (76%), health consequences of refusing a medical intervention (69%), and other methods of treatment (46%). However, patients pointed out a number of problems in physician-patient communication.

**Conclusion:**

Communication practices during informed consent-obtaining process in hospitals in Zagreb are based on a model of shared decision-making, but paternalistic physician-patient relationship is still present. Our results indicate that Croatia is undergoing a transition in the physician-patient relationship and communication.

Good communication between health care professionals and patients ensures good patient-physician understanding, which in turn influences patient satisfaction, compliance, medical outcomes cost-containment, and overall quality of health care (1,2). Effective communication is a prerequisite for the informed consent-obtaining process (3). This process emphasizes collaboration between the physician and patient, and enables patients to co-decide by being given all the information related to their illness, specific diagnostic and therapeutic procedures, as well as alternative treatment procedures (4,5). An analysis of the informed consent process can be a useful tool for understanding the physician-patient communication (6). Although informed consent is a well-accepted practice, it often fails to achieve its purpose, because its implementation is often reduced to signing the consent form (7). For example, Lavelle-Jones et al showed that 69% of patients had not read the consent form before signing it (8). However, in order to make competent decisions, patients need to understand what lies behind their physicians’ recommendations and be able to discuss them. By becoming competent communicators, the patients will be able to actively participate in communication and reach an agreement with their physicians (9). This is the basis of shared decision-making.

The issues of physician-patient communication and informed consent are of great importance for a transitional country like Croatia. In transitional countries, there is often a discrepancy between the actual legal provisions and situation in health care system because of poor implementation of legal standards (10,11). Transitional countries also often display specific cultural patterns of physician-patient communication (12-14), such as the lack of the information exchange and a paternalistic approach to patients (10,12,14). To the best of our knowledge, these issues have not been systematically studied in hospitals in transitional countries of Central and Southeast Europe, except in Slovakia (15).

Since communication between physicians and patients and informed consent process are culturally dependent (14,16,17), we explored physician-patient communication practices that are part of the informed consent-obtaining process. Previous studies done in Croatian hospitals dealt mainly with communication and informed consent process in certain groups of patients (18-20). Frković et al conducted a study on pregnant women, less than half of whom were well-informed about their pregnancy, the status of their child, and complications (18). Kusec et al (19) studied surgical patients and concluded that the patients' conversational style language should be used in the development of written materials for informed consent forms (19). Jukic et al reported that only 12% of surgical patients received enough information to make decisions about treatment and that physicians have better understanding and knowledge about informed consent than patients (20).

The aim of our study was to explore the practices of physician-patient communication during the process of obtaining informed consent in a hospital setting in Croatia. The study included patients from different hospital departments who were able to give consent regardless of their diagnosis because the Croatian Act on the Protection of Patients’ Rights requires informed consent for all patients and for all diagnostic and therapeutic procedures (21).

## Methods

The study was conducted in the period from April to December 2011 with the previously validated Questionnaire on Patient’s Rights, created in the Medical School in Martin, Slovakia by Nemcekova et al (15). As Croatia and Slovakia have similar cultural and political background we believed this questionnaire to be suitable for the Croatian population. The Croatian-language version was obtained by translation and back-translation approach. The first part of the questionnaire contains 32 items, 20 of which deal with physician-patient communication and informed consent process, 6 with physician-patient relationship, and 6 with other patients' rights such as the right to privacy and confidentiality. With certain multiple-choice questions, there is a possibility of making additional comments. The second part of the questionnaire has 12 items on socio-demographic data. Respondents who participated in the study had to be staying in the hospital for more than two days for various diagnostic and/or therapeutic procedures, and voluntarily agree to anonymously fill in the questionnaire before hospital discharge. The study excluded all patients without the capacity to make consent, such as pediatric, psychiatric, and intensive care patients. We decided to include all tertiary level hospitals in the City of Zagreb county. There are several reasons for this. The quality of health care in tertiary level hospitals is expected to be very high due to the complexity of diagnostic and therapeutic procedures. The complexity of information received by patients in the tertiary level hospital can be high, which makes this type of communication especially interesting for our purposes. Also, the City of Zagreb county has two out of five university hospital centers in Croatia, three out of three clinical hospitals, and one out of three clinics (22). Furthermore, according to the data for 2010, Zagreb tertiary level hospitals have the greatest number of physicians (1779 out of 3050 in Croatia), discharged patients (220 000 out of 367 000), beds (5832 out of 9789), and patients referred from other counties (23). Finally, previous research at the tertiary level dealt only with surgical patients mainly in university hospital centers not including patients in clinical hospitals or clinics (18-20).

We sent a request for participation to six hospitals, five of which accepted (University Hospital Sveti Duh, University Hospital for Infectious Diseases Dr Fran Mihaljević, University Hospital Merkur, University Hospital Centre Sestre Milosrdnice, and University Hospital Dubrava). Ethics Committee of these hospitals approved the study. The University Hospital Center Zagreb refused our request with the explanation that they were undergoing an accreditation process. Five departments from each hospital were randomly selected using a computer program (*www.randomizer.org*). All departments were arbitrarily divided into surgical departments (abdominal surgery, cardiac surgery, vascular surgery, neurosurgery, orthopedics, urology, ophthalmology, gynecology, maxillofacial surgery) and internal medicine departments (hematology, cardiology, nephrology, gastroenterology, diabetology, oncology, infectology, dermatology, immunology, and pulmology). Ten questionnaires were collected per each department, which makes 50 questionnaires per each hospital. The total sample consisted of 250 participants.

Quantitative data were analyzed by IBM SPSS Statistica, version 19.0.0.1 (*www.spss.com*). The differences between the category variables were tested using χ^2^ test with Yates correction. Significance level was set at *P* < 0.05. Qualitative data obtained from patients’ comments were not analyzed because only a small fraction of respondents made comments. However, we present them to complement our quantitative data (Supplementary material(supplementary material)).

## Results

The study included 250 patients from five hospitals in Zagreb, with a response rate of 78%. Fifty three percent of all patients were hospitalized at internal medicine departments and 47% at surgical departments. All patients were adults between 18 and 86 years (average age 53.2 ± 16.8 years) (Table 1).

**Table 1:** Socio-demographic characteristics of study patients

	n (%)
Sex:	
female	124 (49.6)
male	123 (49.2)
did not answer	3 (1.2)
Marital status:	
married	151 (60.4)
unmarried	38 (15.2)
divorced	13 (5.2)
widow/er	30 (12.0)
did not answer	18 (7.2)
Education:	
low (0-8 y)	39 (15.6)
medium(9-12 y)	126 (50.4)
high (>13 y)	81 (32.4)
did not answer	4 (1.6)
Place of residence:	
City	182 (72.8)
countryside	61 (24.4)
did not answer	7 (2.8)
Country:	
Croatia	229 (91.6)
Bosnia and Herzegovina	3 (1.2)
Slovenia	1 (0.4)
did not answer	17 (6.8)
Religion:	
Roman Catholic	205 (82.0)
Greek Catholic	2 (0.8)
Orthodox	5 (2.0)
Protestant	1 (0.4)
Jew	1 (0.4)
Moslem	3 (1.2)
other (atheist, agnostic)	18 (7.2)
did not answer	15 (6.0)

Two items of the questionnaire assessed the circumstances during which the patients received information on the names and specialties of their caregivers. Seventy-three percent of physicians and 55% of nurses introduced themselves. Twenty five percent of patients reported that they found out their nurses’ from other patients. However, in some cases the patients’ comments indicated differently (Supplementary material(supplementary material)).

Four items dealt with the patients' right to receive complete information about their health. Seventy eight percent of respondents stated that they received the information about their diagnosis, 64% were informed about possible treatment methods, and 41% about their prognosis. In 85% of the cases, patients stated that the received medical information was complete, understandable, and considerate. The content of the physicians-patient conversation was mostly related to the illness and its nature (74%) (some of the examples include medications that were discussed in 48% of cases, the impact of disease on lifestyle in 38%, nutrition in 32%, physical activity in 28%, change in workload in 20%, and taking liquids in 17%). Ninety seven percent of patients stated that they would like to know the truth about their health condition while being treated in hospital. We did not collect the information about patients’ treatment outcomes. Patients’ comments indicated satisfaction with the received information about their health and trust in health care professionals (Supplementary material(supplementary material)).

Five items dealt with patients' right to receive complete information about recommended examinations and procedures, possible benefits and risks of undertaking or not undertaking the recommended examinations and procedures, and the alternatives to the recommended procedures. Fifty seven percent of patients assessed the obtained level of information as high, 37% as average, and only 5% as low. Patients were informed about health consequences of a refusal in 69% of cases, health risks of the proposed procedures in 76%, health risks of the proposed treatment in 74%, and alternative methods of treatment in 46%.

Three items assessed patients' right to express their opinion and make decisions about the recommended tests or procedures. Eighty-five percent of patients were able to express their views and opinions about diagnostic and therapeutic procedures, and 77% of these views were taken into account. Nine percent of patients were not able to express their opinion. Eighty seven percent of patients could express their approval or refusal of the proposed procedure. For 94% of patients, illnesses, diagnostic procedures, and treatment were discussed in a reasonable and considerate manner. The physicians explained the importance of the proposed diagnostic and therapeutic procedures in 74% of cases, and nurses explained care methods and procedures in 77% of cases. Seventy-six percent of patients always knew the order of diagnostic and therapeutic procedures. In 92% of cases, patients considered that they were notified on time about hospital discharge, and in about half of the cases, further monitoring by general practitioners was suggested. Ten percent of patients answered that they complained when they were not satisfied with medical care. However, some of the comments suggested differently (Supplementary material(supplementary material)).

Five questions dealt with the relationship of health workers and patients. Patients who were present while another patient was dying reported that health care provision was humane and dignified, and patients who were present during another patient’s examination that differed by smell and appearance stated that in most cases such a patient was given adequate care. In 82% of cases, health personnel helped to ease the pain and suffering of patients. However, different patterns of behavior were also observed (Supplementary material(supplementary material)).

When asked about the relationship with the physicians, 98% of patients stated that they had confidence in physicians and respected them. However, patients also observed and complained about some less desirable situations (Supplementary material(supplementary material)).

Patients from surgical departments received higher level of information than those from internal medicine departments, 70.7% vs 46.6%, *P* < 0.001. The potential risks (87.7% vs 65.6%, *P* < 0.001) and other methods of treatment (63.3% vs 34.9%, *P* < 0.001) were discussed more in surgical than in internal medicine departments (Table 2). Patients from different hospitals showed significant differences in responses (Table 2).

**Table 2:** Responses to certain questions among patients from different hospitals and different departments

Question	Percent of patients in hospital					*P†*	Percent of patients in a department		*P^†^*
	SD	FM	M	SM	D		internal	surgical	
Level of received information:									
high	58.0	20.0	61.2	60.4	90.0	<0.001	46.6	70.7	<0.001
average	36.0	68.0	38.8	33.3	10.0		46.6	26.7	
low	6.0	12.0	0.0	6.3	0.0		6.9	2.6	
Were you able to express opinion after receiving information about proposed diagnostic and therapeutic procedures?									
yes, taken into account	87.2	71.7	83.3	78.3	87.8	0.066	74.6	90.0	0.007
yes, not taken into account	4.3	8.7	10.4	6.5	12.2		12.7	3.6	
no	8.5	19.6	6.3	15.2	0.0		12.7	6.4	
Were you able to give your consent or refusal of proposed diagnostic and therapeutic procedures?									
yes, taken into account	95.8	64.4	92.0	89.6	92.0	<0.001	81.0	93.9	<0.010
yes, not taken into account	0.0	6.7	0.0	2.1	6.0		4.8	0.9	
no	4.2	28.9	8.0	8.3	2.0		14.3	5.2	
Were you informed about health consequences of refusal?									
yes	66.7	46.8	77.6	75.0	90.0	<0.001	65.6	78.1	0.032
no	33.3	53.2	22.4	25.0	10.0		34.4	21.9	
Were you informed about the health risks of proposed procedures and examinations?									
yes	77.1	51.0	93.6	82.0	88.0	<0.001	69.5	87.9	<0.001
no	22.9	49.0	6.4	18.0	12.0		30.5	12.1	
Were you informed about the health risks of proposed treatment?									
yes	76.6	54.2	85.4	74.0	89.8	<0.001	65.6	87.7	<0.001
no	23.4	45.8	14.6	26.0	10.2		34.4	12.3	
Were you informed about other methods of treatment?									
yes	49.0	27.1	39.1	47.9	76.6	<0.001	34.9	63.3	<0.001
no	51.0	72.9	60.9	52.1	23.4		65.1	36.7	

*SD – University Hospital Sveti Duh; FM – University Hospital for Infectious Diseases Dr Fran Mihaljević; M – University Hospital Merkur; SM – University Hospital Centre Sestre Milosrdnice; D – University Hospital Dubrava.

† χ^2^ test.

## Discussion

Our results indicated that the communication during the informed consent-obtaining process in hospitals in the City of Zagreb county was based on the model of shared decision-making, but paternalistic relationship is still present. Communication between physicians and patients can be described by different models of physician-patient relationship. The two most frequent models are the paternalistic decision-making model, in which the physician has the dominant role as the decision maker, and the informed decision-making model, in which the physician provides information and patients make decisions on their own (24,25). A third model, required by the Croatian law, is the shared decision-making model (21,24). In this model, the patient and the physician equally exchange information across all stages of the process (25), which is the essence of the informed consent process. The right to information and the right to accept or refuse specific diagnostic procedures are an important feature of this model, as well as two-way communication and the availability of information on other treatment options. Modern medical practice undergoes a transition from a paternalistic approach toward physician-patient partnership (26). Similarly to our study, Gadzek also found changes in the traditional paternalistic relationship in a study on GPs and opioid dependent patients in Croatia (14). However, Talanga (26) found the paternalistic model to be still predominant in Croatia, which is also the case in the low health culture communities in the third world countries.

Our results showed that patients believed that they were adequately informed. Still from patients’ comments, we can see that some of the respondents complained about the superficiality of the process or the lack of adequate information. In fact, every ninth patient stated that he/she received information that was incomplete or not understandable. Barnett et al (27) observed that patients believed that they were well informed despite the fact that they did not receive all the information, eg, about side effects or complications. Patients often feel that they are adequately informed despite their inadequate understanding of the received information or inability to fully recall it (16,28,29). It remains to be answered what should be considered complete information and what the basis of patients’ decision-making is.

Some of the patients’ comments indicated that they considered themselves incapable of being a part of the decision-making process and that it is not the custom to take the patient's opinion into account. Talanga explains this by paternalistic expectations of Croatian patients, left over from the socialist era of free medical care, that physicians must fulfill their duty and help the patient unconditionally (26).

Furthermore, although our study suggests that physicians are actively involving patients in their treatment decision-making, 69% of patients were actually informed about the health consequences of refusing medical intervention and 46% about alternative methods of treatment. This indicates a certain inconsistency in applying shared decision-making model in everyday practice in Zagreb hospitals. The most important factors for patients were two-way communication, information on other treatment options, severe complications of medical intervention, and consequences of not undergoing an intervention, as well as the impact of interventions on future treatment and their long-term impact on patients’ work ability (30). Less important were technical details of the procedure and less severe complications (30).

In our study, potential risks of treatment were more discussed in surgical than in internal medicine departments, and patients from surgical wards received higher level of information. Braddock et al (6) also pointed out that surgeons spent more time informing their patients than general practitioners, and that general practitioners should be more involved in decision-making (31). However, Jukic et al (32) found that internists spent more time informing their patients than surgeons. We did not ask who, when, and how initiated the discussions and this needs to be further investigated.

Patients did not always know health care workers’ names, although introducing oneself is considered to be one of the norms of polite behavior. We also found that respondents displayed great confidence and respect for health care workers, although some patients’ comments indicated minor negative examples.

Ten percent of patients complained when they were not satisfied with health care, which could indicate high levels of satisfaction. It seems that these complaints were addressed in an adequate way although some of the respondents did not think that complaining could improve the situation. Patients also decided to complain to another physician or a nurse, which suggests problems in physician-patient communication.

The quality and the comprehensiveness of the informed consent process was significantly higher in the University Hospital Dubrava than in other hospitals, but these findings need further investigation. In the University Hospital for Infectious Diseases Dr. Fran Mihaljević, health consequences of refusal were discussed with less than half (46.7%) of patients and alternative methods of treatment were discussed in 27.1% of cases, which is significantly lower than in other hospitals. These data can be explained by a specialized function of this clinic in providing specific antimicrobial treatment often with no alternative treatment options. Additionally, patients often arrive to this clinic when their treatment options have been exhausted. These results confirm our results from internal medicine departments.

Our study has certain limitations. We did not record the actual conversations between physicians and patients, but rather investigated patients’ recall of the information received in those conversations. Also, we did not collect the information about patients’ treatment outcomes, although patients with sub-optimal treatment outcomes could have given more negative responses. Finally, no substantial qualitative analysis was done and giving comments was optional.

To get more precise data, further quantitative and qualitative research should be conducted. There were differences in the physician patient-communication practices among certain hospitals, as well as between surgical and internal medicine departments, which indicates a lack of uniformity in the informed consent process. Therefore, we propose the following steps: communication skills training for medical students, residents, and specialists; specification of the information level that patients need to receive; defining by law the catalog of procedures that are a prerequisite for obtaining consent in a written form; analyzing the content and readability of the informed consent forms; further investigation of communicational practices in health care settings in Croatia at all levels of health care systems; and increasing the importance of informed consent procedures within the framework of hospital accreditation in Croatia.
