# Supplementary material for: Are physician-patient communication practices slowly changing in Croatia? – a cross-sectional questionnaire study
Source: Croat Med J. 2013 Apr;54(2):185–91. doi: 10.3325/cmj.2013.54.185 (PMC3641876; doi:10.3325/cmj.2013.54.185)
Supplement: Supplementary material [file CroatMedJ_54_s010.pdf]

Supplementary material 1. Patients' comments

Patients' comments regarding circumstances during which the patients received information on the names and specialties of their caregivers:

"Examinations were performed by various physicians, without anyone ever introducing himself/herself."

"I found out about their names when they were calling each other during the rounds."

We also found the following comment:

"I do not know the names of the nurses, there are a lot of them so I cannot remember all the names."

Patients' comments regarding the patients' right to receive complete information about their health:

"I got the answers to all my questions."

"I learned something new."

"I do not often ask doctors questions, because I trust them, I do not like to bother them."

A patient's comment on the content of his/her conversation with physicians: *"The type of surgery, how it is performed, how I would feel after the surgery, pain, the possibility of taking painkillers."*

Patients' comments about patients' right to receive the complete information about recommended examinations and procedures, possible benefits and risks of undertaking or not undertaking the recommended examinations and procedures, and the alternatives to the recommended procedures, and comments about right to express opinion and make decisions. Comments are divided in two types:

Type 1 (respondents' complaints about the superficiality of the process):

"There was no occasion to discuss these matters."

"Conversations with physicians was very brief and superficial, I had only a few minutes to discuss issues with them during the rounds."

"I just had to keep quiet."

Type 2 (respondents do not think they are capable of being a part of the process).

"I did not express my opinion."

"No, I'm not competent to make or have an opinion about diagnostic and therapeutic procedures; I did what I was told to."

"I do not express my opinion because I do not understand medicine."

"I am a layman and I leave things to experts."

The patients' comments on healthcare professionalism and complaint procedures can be divided in three groups:

Positive views:

"Pleasantly surprised by the professionalism and courtesy, the staff does not look down on patients."

"They were always available."

"Everything was according to schedule and the staff was behaving correctly."

"I complained to the Ministry of Health and now I am more than satisfied."

Neutral views:

"I complained to the nurse."

"I complained to another doctor."

Negative views:

"Is there any point to complain?!"

"I complained at the reception."

"Would it change anything? I do not think so."

Patients' comments regarding the relationship between health workers and patients:

"Nurse Nevenka was arrogant and wicked."

"I have seen physicians very rarely and briefly."

Patients' observation and complain about some less desirable situations when asked about the relationship between physicians nad patients:

"Communication between the two doctors was not good, they disregarded the findings of each another."

"Physician did not want to talk with me sufficiently."

"He got angry if I asked questions."
